# A Method for Detection of Corn Kernel Mildew Based on Co-Clustering Algorithm with Hyperspectral Image Technology

**DOI:** 10.3390/s22145333

**Published:** 2022-07-17

**Authors:** Zhen Kang, Tianchen Huang, Shan Zeng, Hao Li, Lei Dong, Chaofan Zhang

**Affiliations:** School of Mathematics & Computer Science, Wuhan Polytechnic University, Wuhan 430048, China; kang12229@whpu.edu.cn (Z.K.); huangtianchen123@gmail.com (T.H.); lihao@whpu.edu.cn (H.L.); dl1401297186@gmail.com (L.D.); charles0zhang@gmail.com (C.Z.)

**Keywords:** hyperspectral imaging, corn kernel mildew detection, unsupervised redundant clustering algorithm, wavelength band selection

## Abstract

Hyperspectral imaging can simultaneously acquire spectral and spatial information of the samples and is, therefore, widely applied in the non-destructive detection of grain quality. Supervised learning is the mainstream method of hyperspectral imaging for pixel-level detection of mildew in corn kernels, which requires a large number of training samples to establish the prediction or classification models. This paper presents an unsupervised redundant co-clustering algorithm (FCM-SC) based on multi-center fuzzy c-means (FCM) clustering and spectral clustering (SC), which can effectively detect non-uniformly distributed mildew in corn kernels. This algorithm first carries out fuzzy c-means clustering of sample features, extracts redundant cluster centers, merges the cluster centers by spectral clustering, and finally finds the category of corresponding cluster centers for each sample. It effectively solves the problems of the poor ability of the traditional fuzzy c-means clustering algorithm to classify the data with complex structure distribution and the complex calculation of the traditional spectral clustering algorithm. The experimental results demonstrated that the proposed algorithm could describe the complex structure of mildew distribution in corn kernels and exhibits higher stability, better anti-interference ability, generalization ability, and accuracy than the supervised classification model.

## 1. Introduction

Corn is one of the three main grain crops in the world, and its quality plays a critical role in food security. However, due to its high original moisture, large embryo and strong hygroscopicity, corn kernels are susceptible to mildew during storage and transport, which seriously affects their quality and poses threats to the health of humans and animals [1]. Therefore, it is of great significance to develop effective methods to accurately detect and timely monitor mildew in corn kernels to ensure food security.

Most traditional detection methods for corn kernel mildew include artificial sensory evaluation and physicochemical analyses [2,3]. Artificial sensory evaluation is easily affected by subjective factors, such as personal experience and physical conditions, it is challenging to ensure accurate and consistent detection, it requires high investment, and has low efficiency. Although the physicochemical analysis method has high accuracy, it involves using some harmful chemical reagents, which may destroy the original structure of corn kernels and has a long detection cycle and poor repeatability. More importantly, none of the above methods can realize real-time online detection. To improve the detection efficiency, non-destructive detection techniques have been widely used for the rapid detection, screening and grading, quality identification, and safety analysis of foods [4,5]. Non-destructive detection methods commonly used in the detection of corn kernel mildew mainly include near-infrared spectroscopy [6], computer vision [7], and electronic nose [8]. However, the point scanning mode of near-infrared spectroscopy largely limits its detection for samples with the non-uniform distribution of mildew.

Moreover, computer vision heavily depends on external visual acquisition equipment and image processing technology, which are vulnerable to environmental factors. The electronic nose lacks stability and applicability. Hyperspectral remote sensing is an emerging interdisciplinary approach [9,10,11]. Because it can simultaneously reflect ground objects’ spatial and spectral information, it is widely used in geological exploration, urban remote sensing and planning, remote sensing mapping, disaster monitoring, and precision agriculture. In recent years, hyperspectral image technology has gradually become a research hotspot in the non-destructive detection of grains [12]. With hyperspectral imaging, some studies have established non-destructive detection methods for fungal infections in rice, wheat, and maize by [13,14,15]. Yin et al. [16] preprocessed the hyperspectral data of corn kernels with different degrees of mildew infection through multiplicative scatter correction. They used the partial least square regression coefficient to select the characteristic wavelength, and finally used the Fisher discriminant analysis for identification. Yang et al. [17] processed the hyperspectral data using a deep sparse autoencoder, selected feature variables by variable combination population analysis and shuffled frog leaping algorithm, and established classification models of kernel extreme learning machine, extreme learning machine, and support vector machine, respectively. Another study [18] analyzed the maize seed mildew classification with hyperspectral image data obtained by a support vector machine based on the linear kernel, support vector machine based on the quadratic kernel, and BP neural network model, respectively. The current mainstream detection methods of corn kernel mildew generally involve supervised learning, which requires large training samples to establish prediction or classification models. However, it is generally difficult to obtain sufficient labeled samples, resulting in inaccurate estimation of the algorithm’s parameters.

Therefore, it is urgent to study the classification algorithm based on unsupervised learning. However, mildew distribution in corn kernels is usually non-uniform, and the existing unsupervised classification algorithms face two significant problems for pixel-level detection of corn kernel mildew. First, the data with complex structures cannot be well classified. For example, prototype-based clustering algorithms, such as K-means, can only well classify clumpy data. Second, the vast computational cost is unsuitable for rapid online detection applications. Traditional nonlinear clustering algorithms, such as spectral clustering, are based on the similarity matrix for data classification to the realize pixel-level classification and require the calculation of the eigenvalues and eigenvectors of the data matrix, which is very large and proportional to the sample size. To achieve pixel-level hyperspectral image classification of corn kernel mildew, this paper proposes an unsupervised co-clustering algorithm based on multi-center fuzzy clustering (FCM) and spectral clustering (SC). The motivation of the algorithm is that FCM can accurately extract data prototypes, and SC can better describe the distribution of the data structure. This algorithm processes the samples by multi-center fuzzy clustering to describe the distribution of mildew in corn kernels, and then merges the obtained cluster centers by spectral clustering, which can not only maintain the nonlinear structure of redundant cluster centers, but also greatly reduce the computational cost.

The proposed algorithm has two scientific contributions. First, we proposed a co-clustering algorithm that combines fuzzy and spectral clustering to classify the structural hyperspectral image data. Since fuzzy clustering can accurately extract data prototypes and spectral clustering can better describe the distribution of data structure, it can better solve the problems of high dimensionality, noise, and nonlinearity faced by hyperspectral image classification. Second, the current corn kernel mildew detection with hyperspectral images is mainly based on supervised classification methods, such as partial least squares and support vector machines. These methods require large training samples, and their performance heavily depends on the quality of the training samples. In this paper, the unsupervised co-clustering algorithm was used for the first time to achieve good results in detecting corn kernel mildew, which provides a feasible solution for later research on grain quality based on hyperspectral image technology. The experimental results demonstrate that the algorithm can not only describe the complex structure of mildew distribution in corn kernels but also possesses strong stability, anti-interference ability, generalization ability, and high accuracy relative to the supervised prediction or classification models.

## 2. Materials and Methods

### 2.1. Experimental Materials

Corn kernel itself has certain amounts of spores and endospores. Endospores are the dormant form of bacteria, and spores are produced by mildew, which can also produce mildew under natural conditions. The corn kernel samples used in this study were purchased from a farmer’s market in Shanxi Province. Corn kernel samples were placed in a constant temperature (28 °C) and humidity (90%) experimental box for cultivation. After eight days, the samples produced varying amounts of mildew. Then, 60 corn kernels were randomly selected as the experimental materials, arranged into a 6 × 10 matrix on the detection board.

### 2.2. Hyperspectral Acquisition System

**Figure 1:**
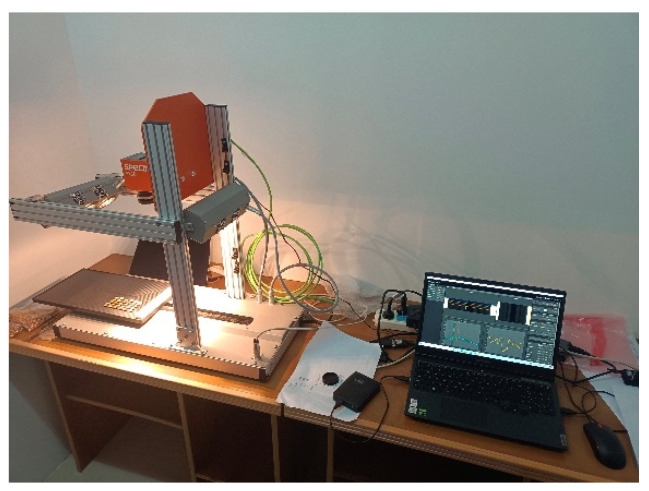
The hyperspectral image data acquisition system of corn kernel mildew.

The hyperspectral image data acquisition system is shown in Figure 1. It comprises a hyperspectral camera (SPECIM FX10, Oulu, Finland), a light source (Osram, two 50 W linear halogen lamps), a mobile platform, and a computer. The imaging method of the hyperspectral imager is linear array stack scanning. The spectral range is 400–1000 nm, the spectral resolution is 5.5 nm, the number of spectral bands is 224, the signal-to-noise ratio is 600:1, and the scanning speed is 65 mm/s. During the image collection, 60 corn kernels were neatly placed on the mobile platform, and the lens was adjusted vertically downward. The system software was used to control the acquisition system to obtain the hyperspectral image data of corn kernels. For the collected hyperspectral images, the ENVI software was used to select the region of interest (ROI), and then the spectral data were analyzed and processed by Matlab.

### 2.3. Data Preprocessing

The data acquisition process by the hyperspectral imaging system is inevitably interfered with by environmental factors, such as noise, background, and dispersive light [19]. The noise information in the spectral data will cause errors in the model established during spectral analysis. Therefore, it is necessary to preprocess the hyperspectral data to remove undesired information, such as uneven illumination, background, and bad pixels. The commonly used pretreatment algorithms include Savitzky–Golay smoothing (SG), standard normal variable (SNV), multiplicative scatter correction (MSC), and first derivative (FD) [20,21]. SG smoothing estimates the average spectral value of a particular wavelength point based on several wavelengths before and after the point with the moving average algorithm or least-squares fitting method, which is then used to replace the original spectral value. Complete wavelength smoothing can eliminate or reduce the spectral data with random noises, but the calculation is complicated. SNV is used for each spectral data point, and by assuming that the absorbance and reflectance values of all wavelength points satisfy the normal distribution, it can perform standard normal treatment on each original reflectance value to eliminate the spectral error caused by surface scattering due to the presence of unevenly distributed granules on the surface. MSC is a spectral data preprocessing algorithm that eliminates scattering caused by uneven particle size distribution. FD can not only effectively remove the noise generated by baseline changes and background interference but also effectively captures the weak information in the sample data. Although derivative preprocessing has numerous positive effects on the spectrum, due to the existence of noise, the random noise in the original spectrum may be amplified, and the signal-to-noise ratio will be reduced without proper derivative preprocessing. Here, to reduce the influence of corn kernel shape and contour on the spectral data and improve the identification accuracy, SNV and MSC were used to preprocess the hyperspectral image data of corn kernel mildew. The calculation formula of SNV is as follows:

(1) $$X_{a,SNV}=\frac{X_{a}-\bar{X_{a}}}{\sqrt{\frac{1}{p-1}\sum_{i=1}^{p}{\left(X_{ai}-\bar{X_{a}}\right)}^{2}}}$$

where $X_{a}$ is the spectral vector of the ath sample, $\bar{X_{a}}$ is the mean value of the spectral vector, and p is the number of wavelength variables.

The calculation formula of MSC is as follows:

(2) $$\{\begin{array}{c} {\bar{A}}_{i,j}=\frac{1}{n}\sum_{i=1}^{n}A_{i,j}\\ A_{i}=m_{i}\bar{A}+b_{i}\\ A_{i\left(MSC\right)}=\frac{A_{i}-b_{i}}{m_{i}} \end{array}$$

In this formula, A represents the n×p dimensional spectral data matrix; n is the number of samples; p is the number of wavelength points used for spectral collection; $\bar{A}$ is the average spectral vector obtained by averaging the original hyperspectral of all samples at each wavelength point; $A_{i}$ is the 1×p dimensional matrix; $m_{i}$ and $b_{i}$ represent the relative offset coefficients and shifts of hyperspectral $A_{i}$ and the average spectral $\bar{A}$ of each sample obtained by unary linear regression.

## 3. Co-Clustering Algorithm

The fuzzy clustering algorithm is one of the current research hotspots in the image segmentation technology. Since fuzzy clustering can accurately extract data prototypes and spectral clustering can better describe the distribution of data structure, an unsupervised classification algorithm is proposed to solve the classification problem in the pixel-level hyperspectral image data of corn kernel mildew. By allowing $X=\left\{x_{1},x_{2},\cdots ,x_{n}\right\}$ to be n data samples, the samples can be divided into c categories with the corresponding cluster centers $\left\{v_{1},v_{2},\cdots ,v_{c}\right\}$; the membership of the jth sample to the ith cluster center is $u_{ij}$, and m is the fuzzy coefficient. The objective function of the proposed co-clustering algorithm is as follows:

(3) $$\{\begin{array}{c} \min J(U,V,Y)=\sum_{i=1}^{c}{\sum_{j=1}^{n}u_{ij}^{m}\left\|x_{j}-v_{i}\right\|}_{2}^{2}+\lambda tr(YLY)\\ s.t.\sum_{i=1}^{c}u_{ij}=1,j=1,2,\cdots ,n,L=I-D^{-\frac{1}{2}}WD^{-\frac{1}{2}} \end{array}$$

where L is the Laplace matrix, $W\in R^{C*C}$ denotes the similarity matrix between the redundant cluster centers, and $w_{ij}=\exp(-\frac{{\left\|v_{i}-v_{j}\right\|}^{2}}{2\sigma^{2}})$ and Y is the classification results of the redundant cluster centers.

By solving the objective function, $U=\left\{u_{ij}\right\}$ and $Y=\left\{y_{jk}\right\}$ can be obtained after the optimization, and the final value of sample $x_{i}$ belonging to the kth class can be expressed as $z_{ik}=u_{ij}\cdot y_{jk}$.

**Figure 2:**
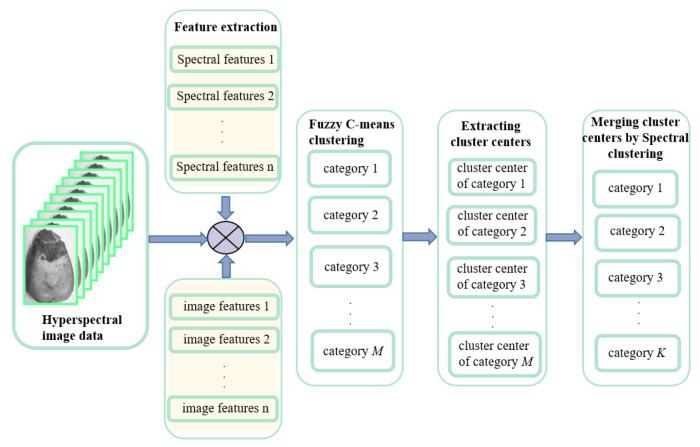
Diagram of the co-clustering algorithm.

**Figure 3:**
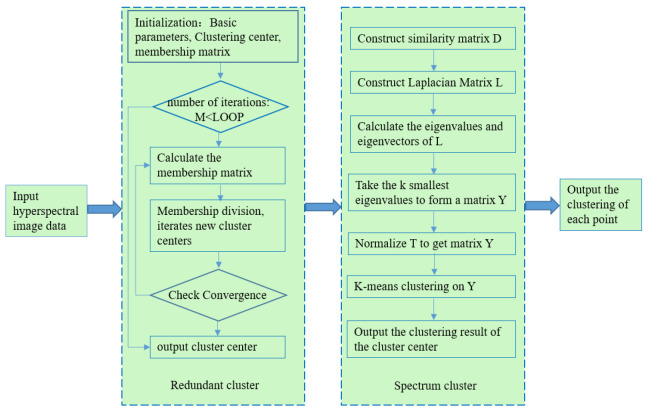
Flowchart of the co-clustering algorithm.

The objective function is solved in two steps to reduce the complexity. First, we used a fuzzy clustering algorithm to handle the preprocessed corn kernel mildew hyperspectral image data, extracted the cluster centers as the next input data, and then used spectral clustering to merge the cluster centers. The schematic diagram of the proposed co-clustering algorithm is shown in Figure 2. As observed from the objective function, the first part of this algorithm implements fuzzy C-means clustering [22,23], and the second part uses spectral clustering [24,25] to merge the redundant cluster centers. Finally, each sample finds the category to which the corresponding cluster center belongs, and the final classification results can be obtained. After the first fuzzy clustering, the vast data set can be compressed. After removing the noises, the obtained cluster centers are processed by spectral clustering. At this time, the amount of data has been dramatically reduced, and the dimensions of the constructed similarity matrix and Laplace matrix will also be sharply decreased. The final clustering results will be more stable, and the calculation will be significantly simplified. The algorithm flow chart is shown in Figure 3.

## 4. Experimental Results and Analysis

### 4.1. Spectral Preprocessing Results

**Figure 4:**
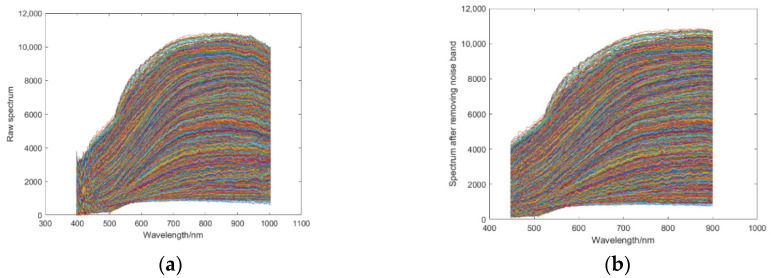
(**a**) Original spectrum of corn kernel mildew (400–1000 nm), and (**b**) spectrum of corn kernel mildew after removal of noisy bands (450–900 nm).

**Figure 5:**
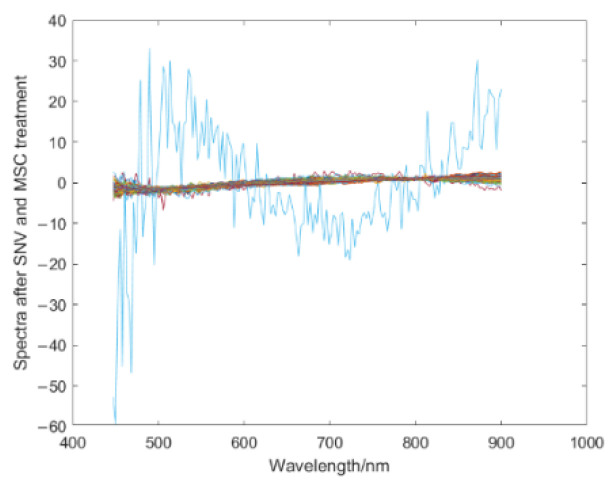
Spectrum pretreated by SNV and MSC.

The scattering phenomenon will occur in collecting spectral data due to the unique morphology of corn kernel surfaces, such as surface granularity and irregularity. To avoid the influence of corn kernel shape and contour on the spectral data and improve the recognition accuracy, the SNV and MSC algorithms were adopted to preprocess the hyperspectral image data of corn kernel mildew. Before the pretreatment, the noises of the front and back bands are considerable during hyperspectral image acquisition, which will harm the post-processing procedures. In the experiment, noisy bands were removed, 168 bands between 450 nm and 900 nm were retained, then SNV and MSC were used for the pretreatment. Figure 4 shows the spectrum of the hyperspectral image data of corn kernel mildew before and after the removal of bands with high noise. Figure 5 shows the spectrum of the hyperspectral image data of corn kernel mildew after SNV and MSC pretreatment.

### 4.2. Gabor Feature Extraction

The two-dimensional (2D) Gabor wavelet is a Fourier transform whose window function is the Gaussian kernel function [26]. It has a high multi-scale and multi-directionality and reasonable resolution in spatial and frequency domains. Two-dimensional Gabor has strong robustness for unfavorable environment conditions, such as illumination, and can effectively characterize the texture features of images. Hence, it is often used in image processing and analysis. In this paper, we extracted the discriminative 2D Gabor texture features of corn kernels. It can remove part of the influence of illumination and noise points on the image quality, as well as make more effective use of the advantages of both spectrum and image of the hyperspectral image data. The expression for a two-dimensional Gabor filter can be defined as follows:

(4) $$\varphi_{\mu ,\nu }(z)=\frac{\left\|k_{\mu ,\nu }\right\|}{\sigma^{2}}\exp(\frac{-{\left\|k_{\mu ,\nu }\right\|}^{2}{\left\|z\right\|}^{2}}{2\sigma^{2}})\left[\exp(ik_{\mu ,\nu }z)-\exp(\frac{-\sigma^{2}}{2}\right)]$$

where μ and ν represent the orientation and scale functions of the 2D Gabor filter, $k_{\mu ,\nu }$ indicates the fundamental frequency vector, which controls the filter’s window size and oscillation frequency, and $z={\left(x,y\right)}^{T}$ represents the coordinate of the pixel location in the image. In this formula, $\exp(\frac{-{\left\|k_{\mu ,\nu }\right\|}^{2}{\left\|z\right\|}^{2}}{2\sigma^{2}})$ is a Gaussian function to reflect the localization information in both the time and frequency domains, σ represents the ratio of the window width to the wave vector length, which is generally taken as σ=2π, and ‖•‖ is a Euclidean parametric operation. Part of the parameters are defined as follows:

(5) $$\{\begin{array}{c} k_{\mu ,\nu }=(k_{v}\cos\varphi_{\mu },k_{v}\sin\varphi_{\mu })\\ k_{\nu }=\frac{k_{\max}}{f^{\nu }}\\ \varphi_{\mu }=\frac{\pi \mu }{8} \end{array}$$

where $\varphi_{\mu }$ is the selection direction for $k_{\mu ,\nu }$, $k_{v}$ is the selection scale of $k_{\mu ,\nu }$, $k_{\max}$ is the maximum frequency, and f is the sample step size. In general, the values of $k_{\max}=\pi /2$,$f=\sqrt{2}$. μ and ν directly impact the completeness of the feature extraction, and the filter set of eight directions and five scales is usually chosen. The 2D Gabor filter defined by the equation can also be written in a complex form as follows:

(6) φμ,ν(z)=Re(φμ,ν(z))+iIm(φμ,ν(z))

Splitting it by the real and imaginary parts gives the following equations:

(7) Re(φμ,ν(z))=‖kμ,ν‖σ2exp(−‖kμ,ν‖2‖z‖22σ2)[cos(ikμ,νz)−exp(−σ22)]

(8) Im(φμ,ν(z))=‖kμ,ν‖σ2exp(−‖kμ,ν‖2‖z‖22σ2)[sin(ikμ,νz)]

When performing 2D Gabor wavelets, the primary 2D Gabor wavelet parameters μ, ν and σ should be set appropriately to obtain a complete image without loss. For the corn kernel image I(z), the Gabor feature extraction is the convolution of this image with the 2D Gabor wavelet function $\varphi_{\mu ,\nu }(z)$.

(9) $$G_{\mu ,\nu }(z)=I(z)*\varphi_{\mu ,\nu }(z)$$

**Figure 6:**
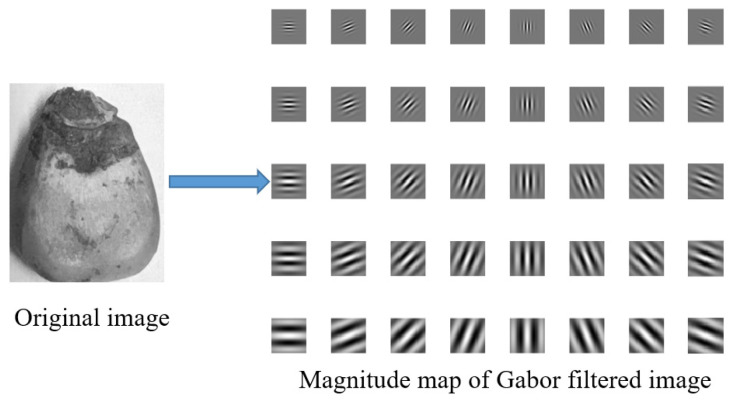
Diagram of Gabor wavelet transformation.

Gabor features are extracted from the image on eight directions μ={0,1,2,3,4,5,6,7} and five scales ν={0,1,2,3,4} to obtain forty sub-outputs. Figure 6 shows the wavelet subgraph obtained from a corn kernel image filtered by 40 Gabor wavelets.

### 4.3. Wavelength Band Selection

Hyperspectral data usually contain spectral bands with redundant and multicollinearity information. Therefore, feature selection of the preprocessed spectral data is critical for improving the model accuracy and reducing the modeling time and complexity. Principal component analysis (PCA) [27] is the most basic algorithm that can effectively solve the problems of hyperspectral data correlation and multicollinearity and achieve feature extraction and data dimension reduction. After the transformation, several new variables, namely principal components (PCs), are used to replace the original variables. While retaining the information of the original variable to the maximum, the primary information is concentrated in the first few unrelated and orthogonal PCs. The basic principle of PCA is to use linear projection to project the original data to the new coordinate system, arrange the PCs according to the data projection variance in turn, and finally obtain the same number of original variables, orthogonal and non-overlapping PCs. Usually, the first few PCs retain the primary information of the original data, and the PCs with less contribution can be ignored. PCA transformation of the original spectral matrix can be described as the following:

(10) Y=XL

where $Y=\left\{y_{1}y_{2}\cdots y_{p}\right\}(p<n)$ is the scoring matrix and $L=\left\{l_{1}l_{2}\cdots l_{m}\right\}$ is the principal component matrix.

**Figure 7:**
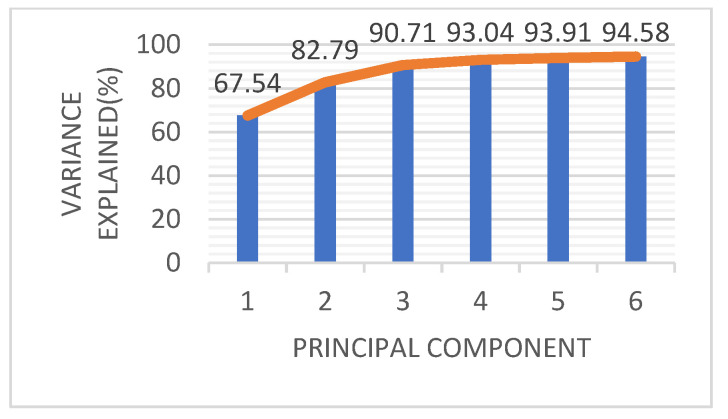
Contribution rates of principal components obtained with PCA feature selection.

In this paper, 94% of the information was retained by PCA. As shown in Figure 7, the accumulative contribution rates of PC1, PC2, PC3, PC4, PC5 and PC6 are 67.54%, 82.79%, 90.71%, 93.04%, 93.91% and 94.58%, respectively. Finally, the data were reduced to 6 dimensions, equivalent to replacing the original 168 dimensions with 6 new dimensions, significantly reducing the amount of data, while retaining the most information in the original data.

### 4.4. Model Identification and Evaluation

**Figure 8:**
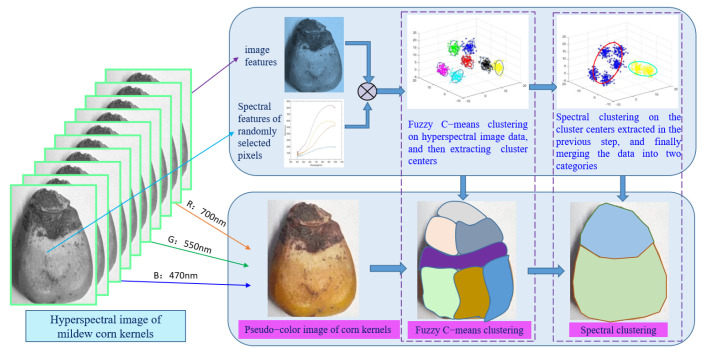
Detection algorithm for the hyperspectral image of corn kernel mildew.

Most noises and redundant data have been removed after pretreatment and feature selection, and the feature data can be obtained after dimension reduction. With the wavelength extracted from PCA as the feature data, the classification model is established and verified by the proposed co-clustering algorithm. The detection algorithm for the hyperspectral image of corn kernel mildew is shown in Figure 8. Since the proposed classification model is an unsupervised learning algorithm, there is no need to divide the samples. In order to better describe the distribution of corn kernel mildew and verify the performance of the algorithm, we made a comparison of the proposed algorithm with some other four representative algorithms with the same data, including the K-means clustering algorithm (K-means), fuzzy C-means clustering algorithm (FCM), kernel fuzzy clustering algorithm (KFCM) and Gaussian mixture model (GMM). In the experiments, all clustering algorithms had the same termination criteria; the fuzzy coefficient in FCM was set as 2; the $K(x_{j},v_{i})$ in KFCM objective function was Gaussian kernel; and the fuzzy weighting index m was set to 2.

**Figure 9:**
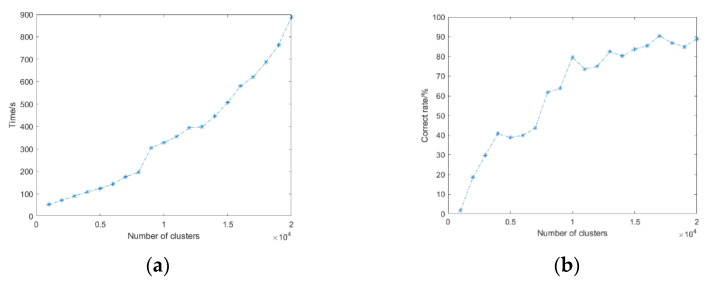
(**a**) Relationship between the cluster number and calculation time; (**b**) relationship between cluster number and accuracy.

The predefined parameters for the co-clustering algorithm include the number for the first and final clustering and the sigma value in the spectral clustering. In order to better describe the distribution of mildew, all data were finally divided into two categories, namely the mildew part and the non-mildew part. Therefore, the final cluster number was set as 2, and the sigma value in spectral clustering was set to 0.5. As for the number of the first clustering, two factors, namely clustering accuracy and computational cost, were considered. Several experiments were designed for in-depth analysis, as shown in Figure 9. A larger number of clustering results in fewer data points in each cluster, and there will be fewer cluster centers in the middle of the two clusters. Therefore, the final clustering effect is better. However, spectral clustering requires the computation of the similarity matrix, and the computing speed is closely related to the amount of data. Hence, the time for calculating each cluster increases with the increasing number of clusters. According to the experimental analysis, the clustering accuracy is the highest when the number of clusters reaches 17,000. Increasing the number of clusters does not significantly impact the results but will greatly increase the calculation time. Therefore, the number of clusters in the following experiment was 17,000.

In addition to accuracy (CR), we combined the normalized mutual information (NMI) and RAND index (RI) to verify the performance of the algorithm jointly. As for the evaluation standards, CR evaluates the efficacy of the clustering by comparing the clustering results with real tag matches; RI is to match the correct and matching error log to evaluate the data; while NMI is mainly different from these two evaluation standards, as it introduces the maximum likelihood estimation and calculation of mutual information and finally normalizes the results.

Currently, most detection methods for corn kernel mildew by hyperspectral image technology only utilize the spectral features and do not take advantage of image features simultaneously acquired by the hyperspectral technology. Hence, we attempted to conduct experiments from two perspectives, including detection only with spectral features and detection with both spectral and image features. 

**Figure 10:**
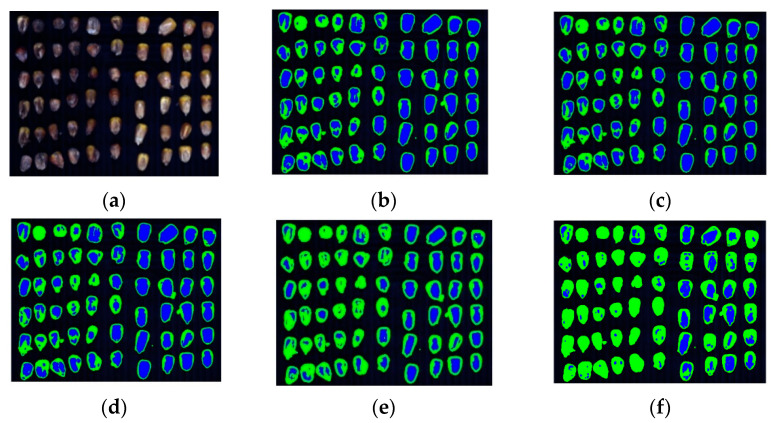
Clustering results in corn kernel mildew hyperspectral image data by different algorithms (spectral features). (**a**), Mildew corn kernels; (**b**), K-means; (**c**), FCM; (**d**), KFCM; (**e**), GMM; (**f**), FCM-SC.

First, the spectral features were clustered, and the clustering results are presented in Figure 10 and Table 1. The mildew part is marked in yellow, while the non-mildew part is marked in blue. The clustering analysis results revealed that due to the non-clumpy and relatively complex structure of the hyperspectral image data of corn kernel mildew, the K-means and FCM based on Euclidean distance had poor performance in NMI, RI, and CR, which were 0.1231, 0.5328, 62.81% and 0.1233, 0.5326, 62.77%, respectively. The GMM algorithm is more suitable for elliptic data, and the clustering results are generally dependent on the cluster center, and thus cannot solve the clustering problem of complex structure data. The KFCM algorithm introduces the kernel function and can map the data to a high-dimensional feature space to obtain more regular data for better clustering. However, the constraint condition of membership sum of 1 makes it sensitive to isolated points and noise. On the other hand, it is an iterative descent algorithm, making it sensitive to the initial cluster center and challenging to converge to the global optima. Therefore, the NMI, RI and CR values are only 0.1232, 0.5325 and 62.76%, respectively. In the proposed co-clustering algorithm, the cluster centers of the fuzzy clustering algorithm are used as the data of spectral clustering, which reduces the sample size from 75,250 to 17,000. If spectral clustering is performed directly on the data, it is necessary to perform eigen decomposition on the Laplace matrix with a size of 75,250 × 75,250, resulting in a high computation cost; thus, spectral clustering cannot be applied to the data. Moreover, because it is difficult for the isolated noise point to become the cluster center, fuzzy clustering also has a certain de-noising effect. The spectral clustering algorithm has excellent clustering performance for non-clumpy data. The NMI, RI and CR values of the FCM-SC algorithm reach 0.5467, 0.8470 and 91.65%, respectively, which are optimal compared with the other algorithms.

**Figure 11:**
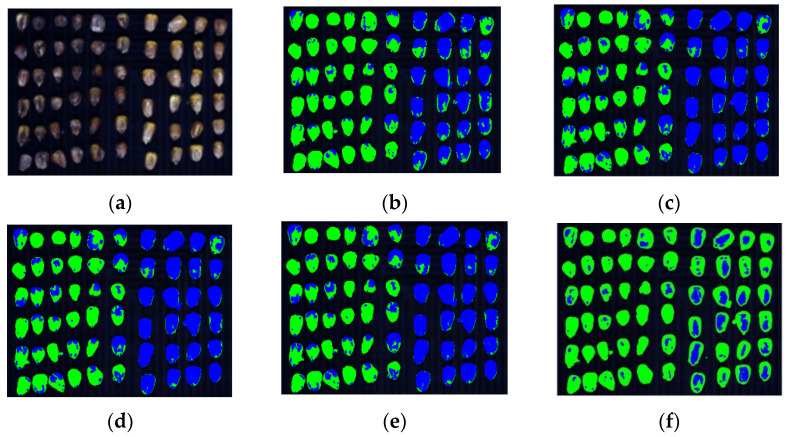
Clustering results in corn kernel mildew hyperspectral image data by different algorithms (spectral and image features). (**a**), Mildew corn kernels; (**b**), K-means; (**c**), FCM; (**d**), KFCM; (**e**), GMM; (**f**), FCM-SC.

Second, detection experiments with both spectral and image features are conducted. Figure 11 and Table 2 show the clustering results after merging the spectral and image features. From a comprehensive perspective, all clustering results have been improved after introducing image features. It is because in addition to the spectral features, Gabor feature extraction is performed on corn kernel hyperspectral images from eight directions and five scales, which means that forty image features are added for each pixel. The accuracy of the proposed algorithm reaches 93.47% and is increased by 2% compared with the accuracy without the addition of the image features, which is the best compared with other algorithms and reflects the algorithm’s stability.

**Figure 12:**
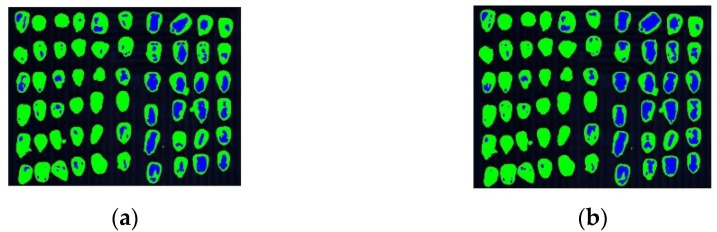
Classification results of SVM and LDA in corn kernel mildew hyperspectral image data (spectral features). (**a**) SVM and (**b**) LDA.

The above results demonstrate that the proposed unsupervised clustering algorithm has excellent performance in the hyperspectral image classification of corn kernel mildew. Currently, supervised classification algorithms are mostly used. We compared the proposed algorithm and the currently used supervised clustering algorithms, namely linear discriminant analysis (LDA) and support vector machine (SVM). Classification results of SVM and LDA in corn kernel mildew hyperspectral image data (spectral features) are shown in Figure 12.

**Figure 13:**
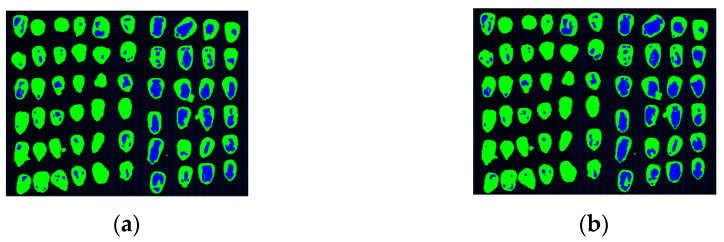
Classification results of SVM and LDA in corn kernel mildew hyperspectral image data (spectral and image features) (**a**) SVM and (**b**) LDA.

**Figure 14:**
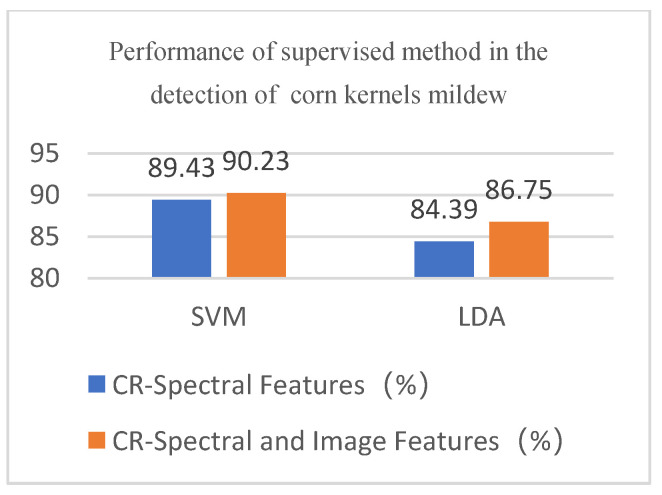
Comparison of the performance of different classification methods.

As shown in Figure 13., the accuracy of SVM and LDA is greatly improved compared with that of the traditional unsupervised clustering algorithm. From the data point of view, the accuracy of the two classification methods has been improved to some extent after the introduction of the Gabor image features. The accuracy rates in the two cases of SVM and LDA are 89.43%, 90.23% and 84.39%, 86.75, respectively, but are still lower than that of the proposed co-clustering algorithm. Comparison of the performance of different classification methods is shown in Figure 14. SVM involves the calculation of a high-rank matrix, and a large sample number will increase the calculation time and space cost. Moreover, SVM is very sensitive to kernel functions and parameters. Although LDA involves a fast calculation, it is unsuitable for non-Gaussian distribution samples due to its linear classification nature. In addition, SVM and LDA are supervised classification algorithms and require labeling the samples and training in advance, while the unsupervised clustering algorithm can perform the classification without sample labeling. 

## 5. Discussion

The experimental results prove that traditional unsupervised clustering methods cannot perform well regarding corn mildew detection. The reason may be that the similarity measure cannot measure the complex data structure well. The supervised classification method is based on a particular training set, and the classification effect is better than that of the traditional unsupervised clustering method. However, the results of the supervised classification method depend on the accuracy of the labels of the prediction set, and the process is complicated. The co-clustering method proposed in this paper can provide more stable and excellent performance to overcome these problems.

The excellent performance of FCM-MC may be attributed to two factors. First, after using the FCM algorithm to cluster the feature data, noises are discarded, and the obtained cluster centers can also better represent the characteristics of the data itself.

Second, the spectral clustering algorithm can cluster in any shape of the sample space and converge to the optimal global solution, and at the same time, the computational complexity of spectral clustering is significantly reduced after the FCM clustering.

## 6. Conclusions

This paper proposes a new corn kernel mildew detection method using hyperspectral imaging. First, SNV and MSC are used for preprocessing to remove the hyperspectral image data with high noise. Then, Gabor features are extracted, and PCA is applied to select the features of the preprocessed data. Finally, an unsupervised clustering algorithm is proposed for pixel-level classification for the first time. The proposed algorithm adopts multi-center fuzzy clustering to describe the distribution of corn kernel mildew and then merges the cluster centers by spectral clustering, which can not only maintain the nonlinear structure of redundant cluster centers but also significantly reduces the computational cost. The experimental results suggest that the proposed algorithm can describe the complex structure of corn kernel mildew distribution with high stability, anti-interference ability, generalization ability, and accuracy compared with the supervised prediction or classification models. At present, the process of redundant clustering and spectral clustering in the algorithm in this paper was carried out in separate stages, which belong to a two-stage clustering algorithm. In the future, we will start from the hyperspectral image characteristics of the corn mildew mechanism and combine redundant and spectral clustering to make the clustering process cooperate to improve the robustness and convergence of the algorithm.

## Figures and Tables

**Table 1:** Performance of different clustering algorithms on hyperspectral data (spectral features).

Method	K-Means	FCM	KFCM	GMM	FCM-SC
NMI	0.1231	0.1233	0.1232	0.1340	0.5467
RI	0.5328	0.5326	0.5325	0.5430	0.8470
CR (%)	62.81	62.77	62.76	64.68	91.65

**Table 2:** Performance of different clustering algorithms on hyperspectral data (spectral and image features).

Method	K-Means	FCM	KFCM	GMM	FCM-SC
NMI	0.1334	0.1453	0.1635	0.1370	0.5885
RI	0.5653	0.5763	0.5854	0.5470	0.8943
CR (%)	64.81	67.85	68.76	64.78	93.47

## Data Availability

The data that support the findings of this study are available upon request from the authors.
